# Construction of a synthetic phage-displayed Nanobody library with CDR3 regions randomized by trinucleotide cassettes for diagnostic applications

**DOI:** 10.1186/s12967-014-0343-6

**Published:** 2014-12-10

**Authors:** Junrong Yan, Guanghui Li, Yonghong Hu, Weijun Ou, Yakun Wan

**Affiliations:** The Key Laboratory of Developmental Genes and Human Disease, Ministry of Education, Institute of Life Sciences, Southeast University, Nanjing, 210096 PR China; Jiangsu Nanobody Engineering and Research Center, Nantong, 226010 PR China; State Key Laboratory of Materials-Oriented Chemical Engineering, College of Biotechnology and Pharmaceutical Engineering, Nanjing Tech University, Nanjing, 210009 PR China

**Keywords:** Nanobody, Trinucleotide cassettes, Human prealbumin, Phage display, Diagnostic application

## Abstract

**Background:**

Nanobodies (Nbs) have proved their great value as therapeutic molecules and clinical diagnostic tools. Although the routine procedure to obtain Nbs is to immunize camels with antigens, it is unavailable to immunize a camel when the antigens are highly toxic, pathogenic or nonimmunogenic. A synthetic phage display library is an alternative to generate Nbs against such targets, besides all the other ones.

**Methods:**

We constructed a large and diverse synthetic phage display Nanobody (Nb) library based on the conserved camel single-domain antibody fragment (VHH) framework of cAbBCII10. Diversity was introduced in the complementarity-determining region 3 (CDR3) by means of randomization of synthetic oligonucleotides. Then human prealbumin (PA) and neutrophil gelatinase-associated lipocalin (NGAL) were used to select specific Nbs from this library. Furthermore, a sandwich enzyme-linked immunosorbent assay (ELISA) was developed to detect PA based on horseradish peroxidase (HRP)-conjugated anti-PA Nb isolated from this study and another biotinylated anti-PA Nb obtained from an immune library, in our previous study.

**Results:**

A large and diverse synthetic phage display Nb library with CDR3 regions randomized by trinucleotide cassettes was constructed. The library size was 1.65 × 10^9^ CFU/mL and the correct insertion ratio was nearly 100%. A Nb against human PA and against NGAL was successfully isolated from the synthetic library. The obtained anti-PA Nb was effectively used to develop a sandwich ELISA for PA detection and it demonstrated a working range from 50 to 1000 ng/mL, with a limit of detection (LOD) of 27.1 ng/mL.

**Conclusion:**

This proposed novel synthetic library was a good source for obtaining some antigen-specific Nbs. This approach could provide crucial support to an immune library and a naïve library in the acquisition of specific Nbs, potentially functioning as a great resource for medical diagnostic applications. In addition, we have successfully developed a novel sandwich ELISA to detect PA, which could provide great assistance for clinical PA detection.

## Background

Since the technique of generation monoclonal antibodies has been discovered, it has showed great value as both diagnostic and therapeutic tool. Monoclonal antibodies are mainly isolated from mouse hydridomas and can recognize the antigens with high specificity and affinity. The basic structure of a conventional immunoglobulin G (IgG) molecule comprises two identical heavy chains and two identical light chains linked together by interchain of disulfide bonds. However, monoclonal antibodies have some limitations. For example, the manufacturing processes in the production capacity of therapeutic antibodies in eukaryotic systems will cause a high cost [1]. Moreover, the large size of monoclonal antibodies may disturb efficient tissue accessibility and penetration [2]. Such problems could be solved by obtaining smaller antigen-binding antibody fragments [3].

With the rapid development of gene engineering techniques, various recombinant antibody fragments, such as antigen-binding fragment (Fab) and single-chain antibody fragment (scFv), have become promising alternatives to monoclonal antibodies [4]. These antibody fragments can maintain the specificity to antigens. More importantly, they are amenable to feasible engineering and of cheaper production as well as they possess some unique properties for various diagnostic and therapeutic applications [5]. A novel type of antibody fragment, termed camel single-domain antibody fragments (VHH) or Nanobody (Nb), is derived from heavy-chain antibodies (HCAbs), which are present in sera of camelids such as camels, llamas, alpacas and dromedaries [6]. With the average size of 15 kilodalton (kDa), Nanobodies (Nbs) are the smallest naturally occurring integral antigen-binding units [7].They are characterized by stringent monomeric performance, high stability, outstanding expression yield in prokaryotic and eukaryotic hosts, and lower immunogenicity [8-10]. Thus, these properties make them advantageous tools for biomedical applications [11-13].

**Figure 1:**
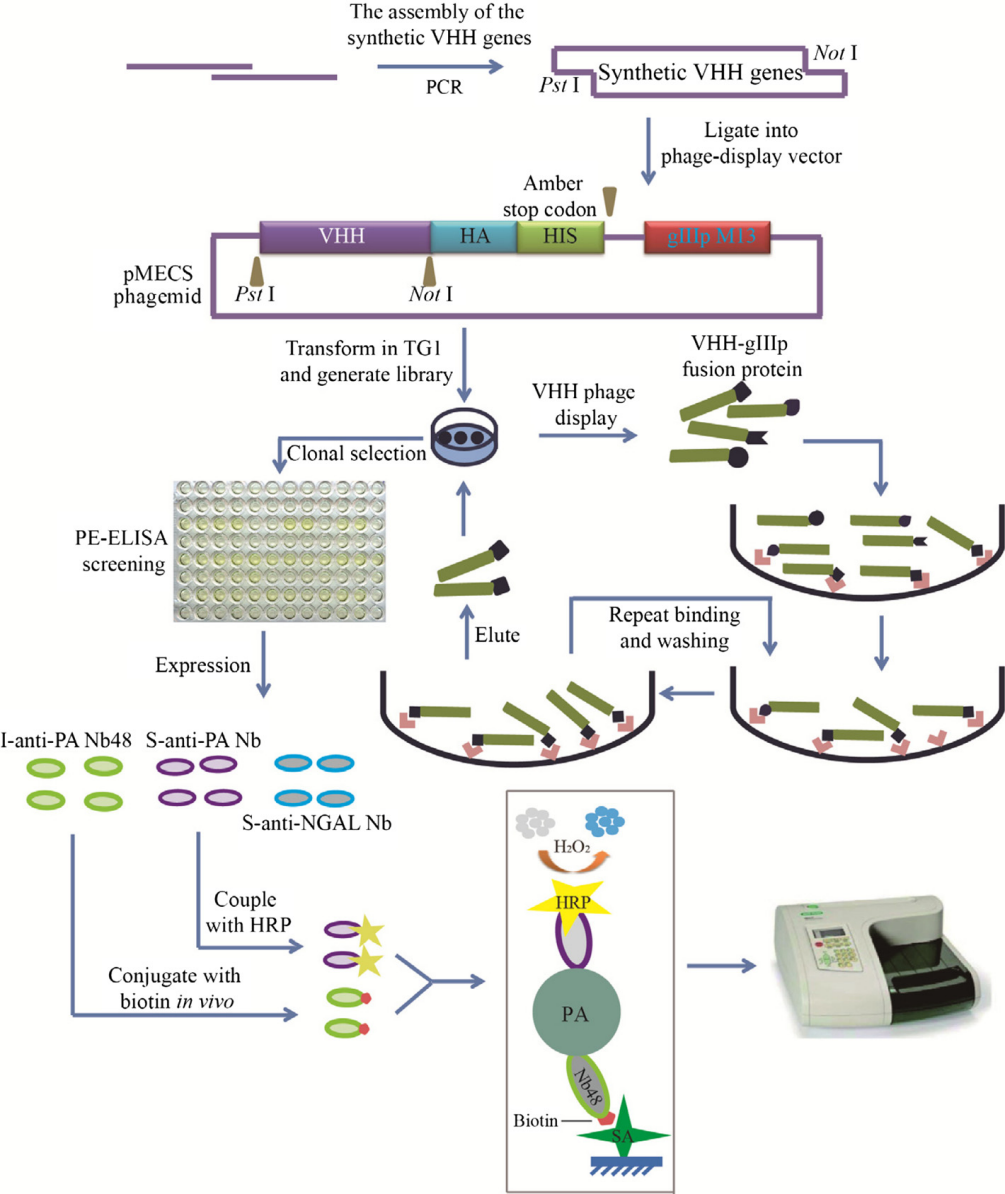
**Sketch map of strategies to construct the synthetic library, select the anti-PA and anti-NGAL VHHs, and detect PA using a Sandwich-ELISA based on the isolated anti-PA Nb and another Nb derived from an immune library.**

Although camelids are large and outbreeding animals, the process to induce HCAbs response in camelids are identical to those used to elicit conventional antibodies in other mammals. Cloning VHH genes and isolation of Nbs from an immunized camelid is a simple and straightforward process. Phage display is the most popular method for camelids Nb library construction. Nb libraries generated from immunized camelids can maintain full functional diversity. Although high affinity Nbs can be isolated in a short period of time, in some cases it is difficult to achieve, for example, when the immunization of camels would be impossible due to a high toxicity or pathogenicity from the antigens, or in another instances if the antigens would produced as inclusion bodies with wrong folded proteins, self-immune antigens and some nonimmunogenic small molecular compounds [14,15]. In order to solve these problems, naïve or synthesized libraries could be an alternative way [16]. Naïve libraries have been reported in several studies with sizes within 1.6 × 10^5^ to 5 × 10^9^ colony-forming units (CFU)/mL [15,17-19]. In this study, as shown in Figure 1, we have successfully constructed a synthetic phage display library by randomizing the complementarity-determining region 3 (CDR3) of Nbs by using a novel trinucleotide cassettes technology. The size of the synthesized library is 1.65 × 10^9^ CFU/mL with great quality and diversity. After several rounds of bio-panning, Nbs against human prealbumin (PA) and neutrophil gelatinase-associated lipocalin (NGAL) were isolated and efficiently expressed, we termed them S-anti-PA Nb and S-anti NGAL Nb, respectively. PA, a hepatic protein, is especially sensitive to early phases of reduced nutrition, particularly caloric intake and nitrogen balance [20,21], in addition, the concentration of PA rapidly returns to normal values within the reference interval, once the malnutrition has been corrected [21]. Therefore, the assay of circulating PA has revealed to be clinically valuable in the evaluation of nutritional status, both as an initial screen and in the monitoring of nutritional recovery [22]. NGAL belongs to the lipocalin superfamily, which is found in granules of neutrophils [23,24]. Several studies have shown that NGAL is an early diagnostic biomarker for acute kidney injury (AKI) in common clinical AKI scenarios including contrast nephropathy, cardiac surgery, critical care and transplantation [25-27]. Herein, by comparing the anti-PA Nb from an immune library constructed in our previous study [28] (we termed them I-anti-PA Nbs), the S-anti-PA Nb showed comparable binding affinity and stability. In addition, based on the use of S-anti-PA Nb coupled with horseradish peroxidase (HRP) and I-anti-PA Nb48 conjugated with biotin, an enzyme-linked immunosorbent assay (ELISA) method to detect PA was developed with a detection range from 50 to 1000 ng/mL with an acceptable correlation coefficient (R^2^) of 0.9592 and a limit of detection (LOD) of 27.1 ng/mL. All together, such a synthetic library represents a good source for isolation of some antigen-specific Nbs in some special cases, providing essential support for both immune library and naïve library, which may be a great resource for various medical diagnostic applications.

## Methods

### Reagents and instruments

NGAL and PA were purchased from Huibiao Biological Technology Co., Ltd. (China). HRP, goat alkaline phosphatase (AP)-conjugated anti-mouse IgG, isopropyl β-D-1-thiogalactopyranoside (IPTG), Bis (p-nitrophenyl) phosphate (BNPP) and 3, 3′, 5, 5′-Tetramethylbenzidine (TMB) were purchased from Sigma-Aldrich (USA). Mouse anti-HA tag antibody was obtained from Covance (USA). *Pst* I, *Not* I, *Nco* I and *BstE* II were obtained from NEB (USA). NI-NTA Superflow Sepharose column was purchased from Qiagen (Germany). Streptavidin (SA) Mutein Matrix was purchased from Roche (Switzerland). Ultra-filtration column was purchased from Millipore (USA). Quick Gel Extraction Kit was obtained from Axygen (China). 96-well Maxisorp plate was purchased from Thermo Scientific NUNC (Denmark). PBS, NaHCO_3_, H_2_SO_4_, NaIO_4_, NaBH_4_, NaCl, MgCl_2_, tryptone, yeast extract, polyethylene glycol (PEG) 6000, D-biotin, tween-20, bovine serum albumin (BSA), ampicillin, kanamycin, imidazole, glycerol, ethylene glycol and glucose were obtained from Sangon Biotech (China). 24-well cell culture plate was purchased from Corning (USA). BeaverNano™ SA Matrix Coated 96-Well Plate was provided by Beaver (China). VCSM13 helper phages, TG1 cells, WK6 cells, plasmids pBAD and pBirA were provided by Serge Muyldermans (Laboratory of Cellular and Molecular Immunology, VUB-Vrije Universiteit, Brussel, Belgium). All aqueous solutions were prepared with deionized water (DI water, 18 MΩ/cm, Milli-Q, Millipore). Absorbance determination was performed on Bio-Rad iMark™ (Bio-Rad, USA). Concentration measurements of mRNA, DNA and protein were carried out with Nano Drop 2000 (Thermo Scientific, USA). Optical density (OD) determination was performed on UV-1800PC spectrophotometer (Mapada, China). DNA was sequenced by Nanjing Springen Biotechnology Co., Ltd.

### Library construction

**Figure 2:**
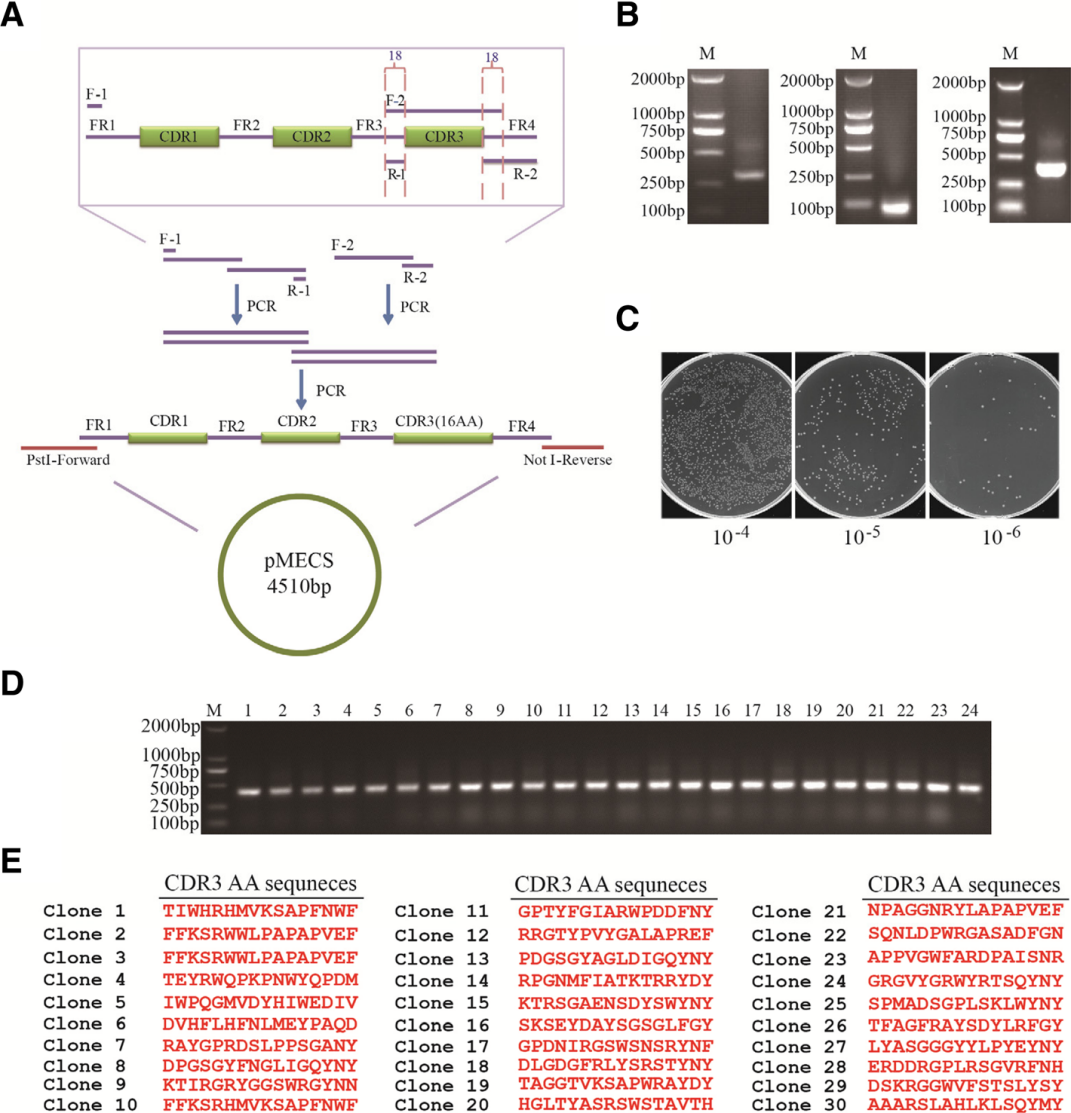
**Construction of the synthetic library. (A)** Schematic flow chart for the assembly of synthetic diversity in CDR3 based on an identified universal VHH framework of cAbBCII10. Synthetic VHH genes were generated by overlap PCR extension. Final PCR products were digested with *Pst* I and *Not* I, gel-purified sequentially and cloned into the phagemid vector pMECS. **(B)** *Left*. F-1 and R-1 were used to amplify a fragment of ~ 300 bp with a determined DNA sequence of cAbBCII10 FR1-FR3 as the template; *Middle*. Agarose gel electrophoresis of the PCR products using F-2 and R-2 as the amplification primers; *Right*. Final products assembled and amplified by overlapping PCR extension. **(C)** The capacity of the library measurement. Scraped colonies were diluted into 5 mL of PBS and 50 μL were used to do gradient dilution. **(D)** 24 clones were randomly picked to estimate the correct insertion rate by performing PCR. **(E)** 30 clones were randomly chosen for sequencing to detect the diversity of CDR3.

This antibody library was constructed based on an identified universal VHH framework of cAbBCII10 [29] with synthetic diversity in CDR3. The diversity of CDR3 was introduced by randomizing the library oligonucleotide DNA through the use of the degenerate codon NNK (N means a 25% mix each of adenine, thymine, cytosine and guanine nucleotides; and K stands for a 50% mix each of thymine and guanine nucleotides) in the library DNA construction with exactly sixteen amino acids (AA) but cysteine (Cys) and stop codon-free. Determined DNA sequence of cAbBCII10 framework region 1 (FR1)-framework region 3 (FR3) was 5′-CAA GTT CAA TTG GTT GAA TCT GGT GGT GGT TCT GTT CAA GCT GG TGG TTC TTT GAG ATT GTC TTG TAC TGC TTC TGG TGG TTC TGA ATAT TCT TAT TCT ACT TTT TCT TTG GGT TGG TTT AGA CAA GCT CCA GGT CAA GAA AGA GAA GCT GTT GCT GCT ATT GCT TCT ATG GGT GGT TTG ACT TAT TAT GCT GAT TCT GTT AAA GGT AGA TTT ACT ATT TCT AGA GAT AAT GCT AAA AAT ACT GTT ACT TTG CAA ATG AAT AAT TTG AAA CCA GAA GAT ACT GCT ATT TAT TAT TGT GCT GCT-3′, which was used to design primers for PCR amplification. The synthetic nucleotides were assembled and amplified by overlapping PCR extension, as illustrated in Figure 2A. The PCR-related primers were: Forward primer-1 (F-1), 5′-CAT ATG CAA GTT CAA TTG GTT GAA-3′; Reverse primer-1 (R-1), 5′- AGC AGC ACA ATA ATA AAT -3′; Forward primer-2 (F-2), 5′-ACT GCT ATT TAT TAT TGT GCT GCT [N]_16_ TGG GGT CAA GGT ACT CAA-3′; Reverse primer-2 (R-2), 5′-GAA TTC CTA AGA AGA AAC AGT AAC TTG AGT ACC TTG ACC CCA-3′, as displayed in Table 1. The final PCR products were digested with *Pst* I and *Not* I and gel re-extracted using a Quick Gel Extraction Kit. The purified PCR fragments were cloned into the phagemid vector pMECS and electro-transformed into *Escherichia coli* (*E. coli*) TG1 cells. The VHH library was grown overnight at 28°C on the plates containing solid 2 × tryptone and yeast extract growth medium (2 × TY medium), which was supplemented with 100 μg/mL ampicillin and 2% (w/v) glucose. The library capacity was measured by counting the number of colonies after gradient dilution. Meanwhile, 24 individual clones were randomly chosen to determine the correct insertion rate by PCR amplification and another 30 clones were sent for sequencing to determine the amino acids distribution and composition of CDR3. Finally, colonies were scrapped from the plates and stored in 2 × TY medium with 1% glucose and 50% (v/v) glycerol at −80°C until further use.

**Table 1:** **The list of primers used in this study**

**Primer**	**Sequence**
F-1	caagttcaattggttgaa
R-1	agcagcacaataataaa
F-2	atttattattgtgctgct[N]_16_tggggtcaaggtactcaa
R-2	agaagaaacagtaacttgagtaccttgacccca

### Selection of anti-PA and anti-NGAL Nbs

500 μL of the library stock was grown in 100 mL of 2 × TY medium containing 100 μg/mL ampicillin, 70 μg/mL kanamycin and 1% glucose. The cultures were grown at 37°C for 2.5 h and then 1 × 10^12^ VCSM13 helper phages were added. After standing for 30 min at 25°C, the cells were centrifuged at 3000 g for 10 min to recover, and the bacterial pellets were added into 300 mL of 2 × TY medium containing 100 μg/mL ampicillin and 70 μg/mL kanamycin. The cultures were incubated at 37°C overnight in a shaker. The VHH library was expressed on the phages after infection with VCSM13 helper phages. The phages were further precipitated with PEG 6000, 0.5 M NaCl and dissolved in PBS. Afterwards, 100 μL of the PBS solution containing 2 × 10^11^ phages were used in screening on immobilized PA and NGAL coated on 96-well Maxisorp plate. Phages expressing anti-PA and anti-NGAL VHHs were constantly enriched via several consecutive rounds of panning. 95 randomly selected individual colonies were cultured in terrific broth (TB) medium containing 100 μg/mL ampicillin in 24-well cell culture plates. The expression of Nbs was induced with 1 mM IPTG followed by an incubation overnight. Periplasmic extraction ELISA (PE-ELISA) was performed to identify the *E. coli* TG1 colonies expressing anti-PA and anti-NGAL Nbs. Finally, the positive colonies were sequenced.

### Expression and purification of isolated Nbs

Plasmids extracted from the positive colonies were electro-transformed into *E. coli* WK6 cells. The recombinant Nbs were produced in shaker flasks by growing the cells in 330 mL of TB medium containing 100 μg/mL ampicillin, 2 mM MgCl_2_ and 0.1% glucose. 330 μL of IPTG (1 M) was added into the medium when the OD 600 was between 0.6-0.9, then the cultures were grown for 16 h at 28°C. Periplasmic extracted proteins were released according standard protocols [30]. The recombinant Nbs were purified by NI-NTA Superflow Sepharose column, sequentially eluted with 100, 250 and 500 mM imidazole solution. Protein purity was verified by sodium dodecyl sulfate polyacrylamide gel electrophoresis (SDS-PAGE), and the eluted fragments were concentrated to 1 mg/mL using Ultra-filtration column and dialyzed into PBS.

### ELISA mapping of anti-PA and anti-NGAL Nb

The Nbs recognizing PA or NGAL were examined by ELISA. Briefly, PA and NGAL were diluted into different concentrations ranging from 0.1 to 20 μg/mL and coated onto 96-well Maxisorp plates in 100 μL of NaHCO_3_ (0.1 M, pH 9.5) at 4°C overnight. After thorough washing with PBST (PBS with 0.05% Tween-20) for 3 times, 300 μL of 3% (w/v) BSA in PBS was added to each well for blocking and incubated for 2 h at room temperature (RT). The plates were washed 5 times with PBST before the addition of 5 μg/mL Nbs followed by an incubation of 1 h. After 5 washes, mouse anti-HA tag antibody (diluted 1:2000 in PBS) was added for 1 h at RT, and then 100 μL of goat AP-conjugated anti-mouse IgG diluted 1:2000 in PBS was added and incubated for 1 h at RT. The catalytic reaction of AP was visualized by using BNPP substrate. After 10–20 min, the readings at 405 nm were measured with Bio-Rad imark™.

### Comparison of reactive sensitivity to PA

The reactive sensitivity comparison between S-anti-PA Nb, I-anti-PA Nb3 and I-anti-PA Nb48 was determined by ELISA. 100 μL of PA (5 μg/mL in 0.1 M NaHCO_3_, pH 9.5) was coated onto 96-well Maxisorp plate overnight at 4°C. 100 μL of the exactly same NaHCO_3_ solution was used as the coating buffer as control. Non-specific binding sites were blocked by 3% (w/v) BSA in PBS for 2 h, the three Nbs were diluted to the same concentration from 0.001 to 10 μg/mL, and were added to the wells followed by incubation for 1 h at RT. The following steps were the same as that described above in ELISA mapping.

### Thermostability analysis

Thermostability was analyzed by ELISA. S-anti-PA Nb, I-anti-PA Nb3 and I-anti-PA Nb48 were diluted to 1 mg/mL in PBS and incubated at 37°C for 0, 2, 12, 24, 48, 60 and 72 h. After incubation, the Nbs were diluted to a final concentration of 10 μg/mL before adding to an ELISA plate coated with 10 μg/mL PA. The rest steps were same as that described above in ELISA mapping.

### HRP coupling

To determine whether the S-anti-PA Nb and I-anti-PA Nbs can recognize the different antigen epitopes, HRP was used to couple to the Nbs. Briefly, 100 μL of fresh NaIO4 (0.1 M) was incubated with 200 μL of 5 mg/mL HRP solution for 30 min at 4°C. Next, 100 μL of 2.5% (v/v) ethylene glycol were added and incubated for 30 min at RT. Then 1 mL of PA-specific Nbs (1 mg/mL) was added and 1 M CB (carbonate and bicarbonate) solution (pH 9.5) was used to adjust the pH to about 9.0, then the mixture was incubated at 4°C overnight in the dark. Afterwards, 20 μL of NaBH4 (5 mg/mL) were mixed into for 3 h at 4°C and finally, the mixture was transferred to Ultra-filtration column to replace the buffer with PBS.

### Assessment of Nbs binding to distinct epitopes on PA

Epitope sharing between the anti-PA Nbs was investigated by a competition ELISA. For this purpose, 96-well Maxisorp plate was coated with 100 μL of 10 μg/mL Nbs in NaHCO_3_ (0.1 M, pH 9.5) at 4°C overnight. After washing with PBST and blocking with 3% BSA, 100 μL of PA (2 μg/mL) was added to the PA-coated wells and PBS without PA was added into the corresponding wells as the control. After 1 h, 5 different Nbs coupled with HRP (2 μg/mL) were added to each Nb-coated well. After extensive washes by PBST, 100 μL of TMB solution was added and incubated for 5–10 min at RT. The enzyme reaction was stopped by adding 50 μL of 2 M H_2_SO_4_ and the absorbance was read at 450 nm.

### Nb biotinylation

The biotinylation of Nb was performed according to our previous studies [31]. Briefly, genes encoding I-anti-PA Nb48 were sub-cloned into plasmid pBAD17 by using *Nco* I and *BstE* II as restriction sites and the recombinant plasmid was co-transformed into WK6 cells with plasmid pBirA. The cells were cultured in TB medium and induced with 1 mM IPTG to express proteins followed by the addition of 50 μM biotin. Biotinylated I-anti-PA Nb48 (BiNb48) was purified by SA Mutein Matrix with 6 mM biotin elution buffer. The residual biotin molecules were removed by Ultra-filtration column with PBS for several times.

### PA detection by ELISA based on biotin-SA interaction

100 μL of BiNb48 (1 μg/mL) in PBST were coated on BeaverNano™ SA Matrix Coated 96-Well Plate at RT for 1 h. After washing with PBST for 5 times and blocking with 5% BSA for 1 h, the wells were incubated with serial dilutions (0, 1, 5, 10, 50, 100, 250, 500, 750, 1000, 2000 and 3000 ng/mL) of PA for 2 h. 100 μL of S-anti-PA Nb coupled with HRP (S-anti-PA Nb-HRP) (1 μg/mL) in PBST containing 5% BSA was added. The rest of the experiment was performed as described above in epitope mapping.

## Results

### Construction of a synthetic phage display Nb library

cAbBCII10 has been proved to be an excellent candidate as an universal framework based on its good stability, good expression level and functional property in the absence of the conserved disulfide bond [29]. Herein, overlapping PCR approach was used to orderly link all oligonucleotides (Figure 2A). The library diversity was dependent on the diverse composition of AA in the CDR3. Assembly PCR was performed with primers listed in Table 1. Individual PCR products were checked by agarose gel as shown in Figure 2B. A total of 50 electro-transformations were performed to obtain satisfactory diversity and the final library was calculated to contain about 1.65 × 10^9^ independent clones (Figure 2C). PCR analysis revealed that 100% of randomly picked clones have been correctly inserted (Figure 2D). Meanwhile, the AA distribution and composition in CDR3 was in good agreement with the designed pattern. Neither stop codon nor Cys was observed in all 30 clones (Figure 2E), which indicated a randomization has been successfully achieved. Most importantly, among these 30 clones, CDR3 showed high diversity, only two of them (Clone 1 and Clone 2) shared the same AA sequence (Figure 2E).

### Bio-panning of synthetic library against PA or NGAL

**Figure 3:**
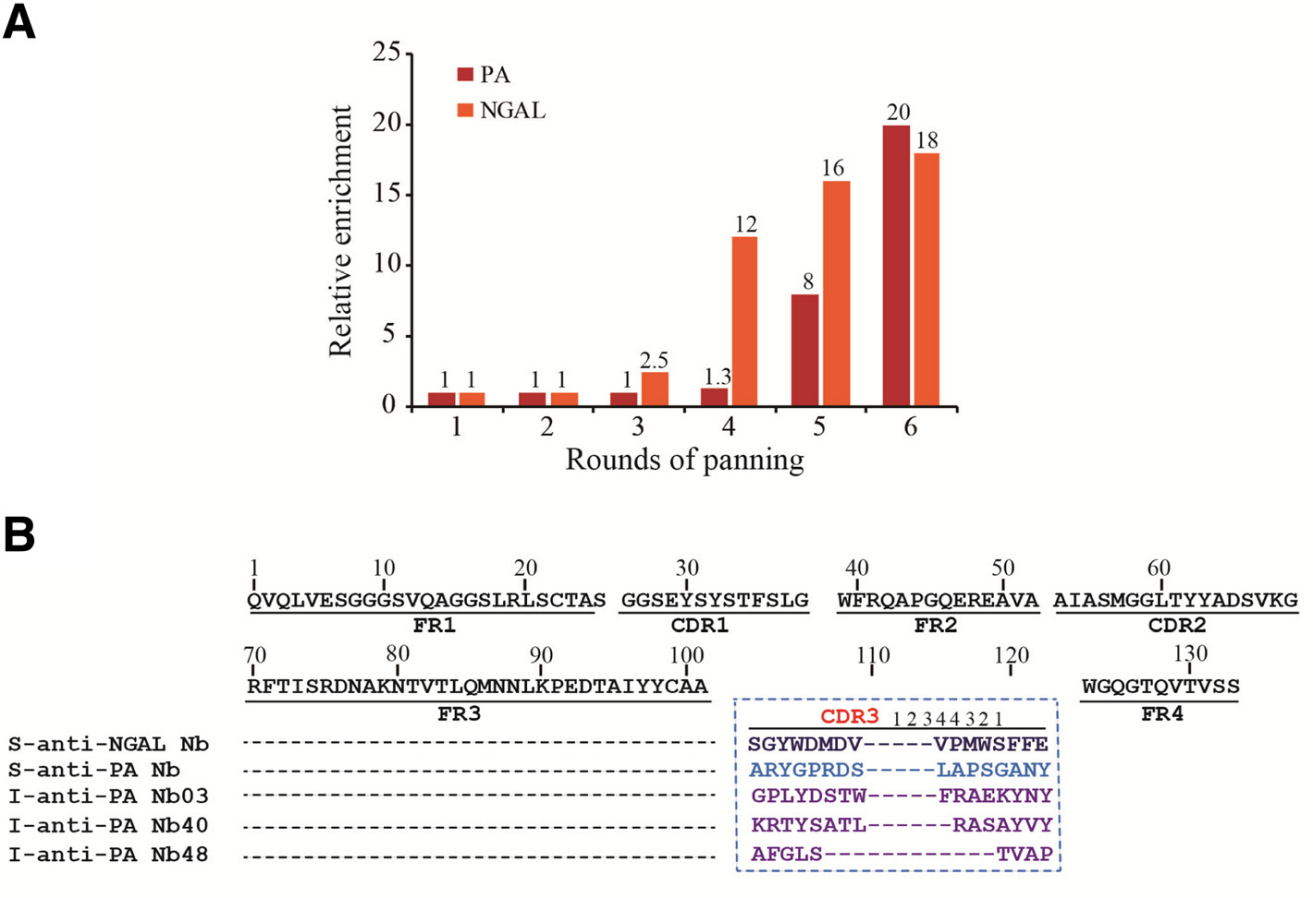
**Selection of anti-PA and anti-NGAL Nbs. (A)** Enrichment exhibition of six rounds by panning. The histogram shows the enriching times for every round of panning. **(B)** The complete AA sequence of selected S-anti-PA Nb and S-anti-NGAL Nb and the CDR3 AA sequence of three I-anti-PA Nbs selected from the immune library.

The synthetic library was used to panning against PA and NGAL. To calculate the enrichment during the course of screenings, we compared the ratio of colony number between PA or NGAL and NaHCO_3_ (0.1 M, pH 9.5) control. As shown in Figure 3A, the ratio was increased to 20 after the sixth round for PA and increased to 18 for NGAL, respectively. Total 15 positive colonies for PA and 54 positive colonies for NGAL with a binding ratio more than 2 have been identified (data not shown). After sequence analysis, the 15 anti-PA clones were classified as one Nb and the 54 anti-NGAL clones were also classified as one Nb as they exhibited the same AA sequence in CDR3. The AA sequences of the two Nbs did not show Cys as expected (Figure 3B). In our previous study, we isolated several anti-PA Nbs from an immune library, which were successfully used to develop a Nb-based flow injection chemiluminescence immunoassay for sensitive detection of PA [28]. The AA sequence comparison demonstrated that they did not share any sequence similarity in CDR3 (Figure 3B).

### Production of soluble Nbs and ELISA mapping

**Figure 4:**
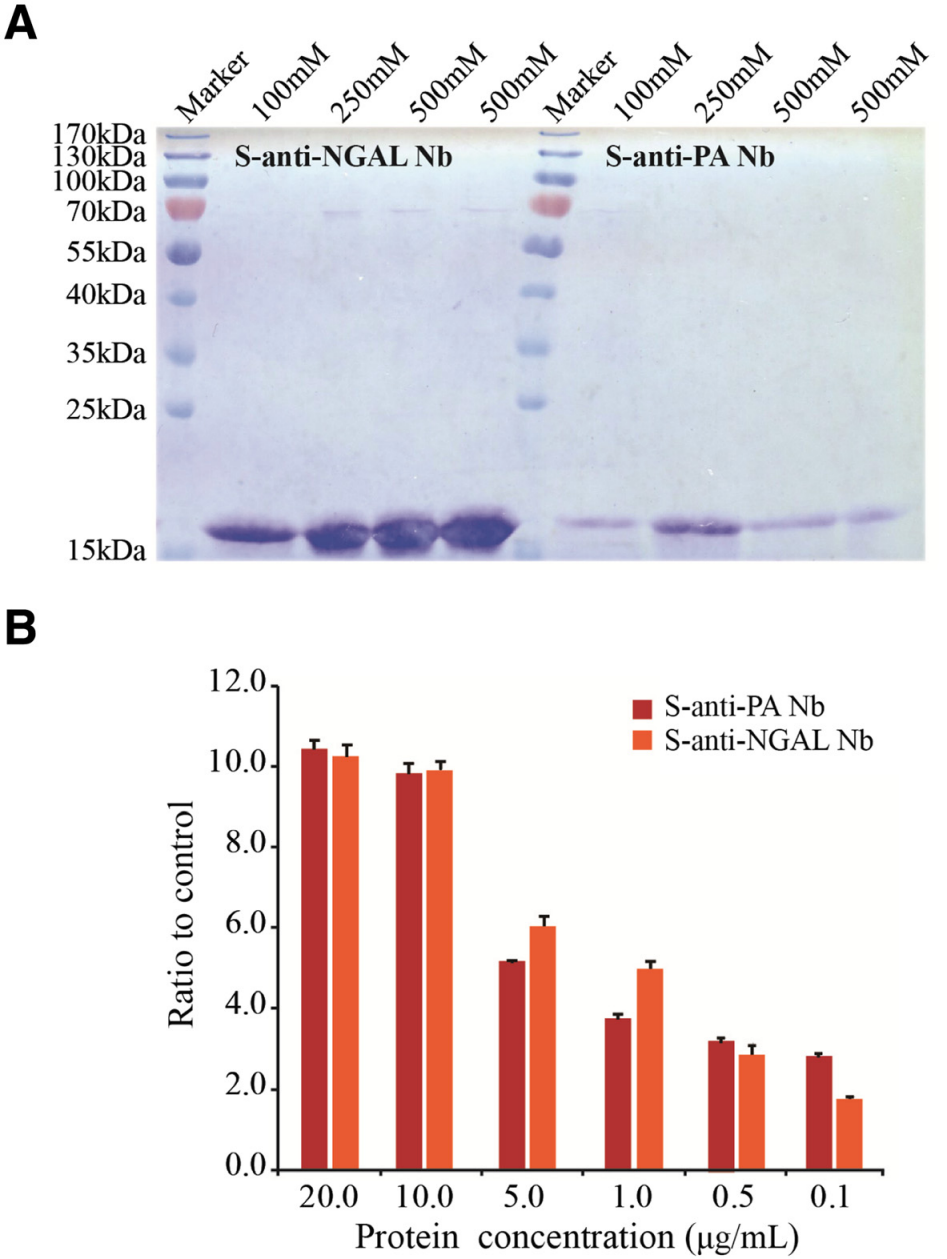
**Nbs purification and ELISA mapping of the purified Nbs. (A)** The recombinant Nbs were orderly eluted with 100, 250 and 500 mM imidazole solution, and the purity was verified by SDS-PAGE. **(B)** ELISA mapping of PA-specific Nb and NGAL-specific Nb using different PA and NGAL concentrations ranging from 0.1 to 20 μg/mL. Three independent experiments were performed.

In order to express the isolated Nbs, the phagemid pMECS was transformed into *E. coli* WK6 cells. These cells have specific properties which cannot suppress the amber stop codon between VHH and the gene III in phagemid pMECS. The induced Nbs were extracted from the periplasmic parts and sequentially eluted with different concentrations of imidazole. SDS-PAGE analysis demonstrated that the purity of Nbs reached 90%, and the production of S-anti-NGAL Nb was much higher than S-anti-PA Nb (Figure 4A). To identify the binding specificity after the pure Nbs were successfully isolated, indirect ELISA was performed to detect the specific interaction between the Nbs and antigens. With the increased concentrations of PA and NGAL, the binding ratio relative to BSA (negative control) was also increased (Figure 4B).

### Nb reactivity to PA by ELISA assay and thermostability analysis

**Figure 5:**
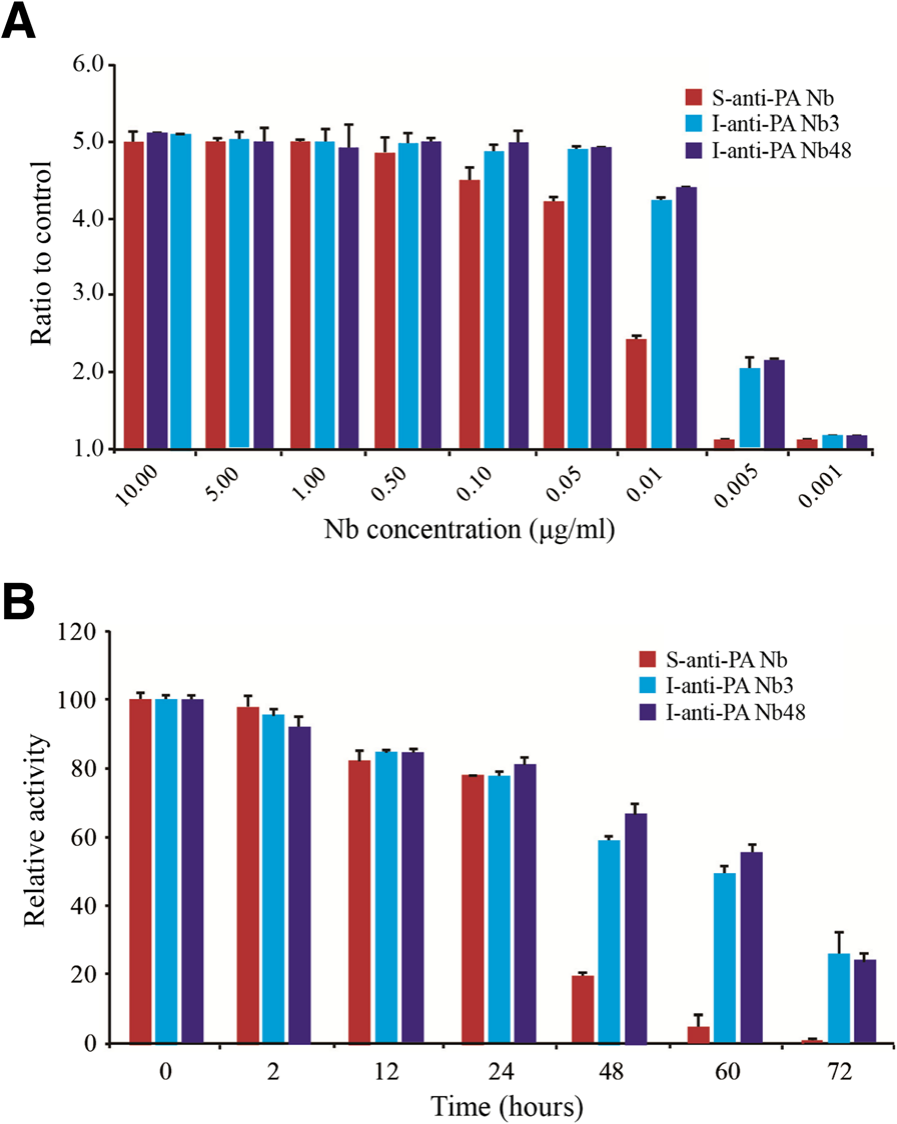
**Comparison of the reactive sensitivity to PA and thermostability analysis of two libraries-derived anti-PA Nbs. (A)** Indirect ELISA was used to compare the reactive sensitivity to PA with different gradient concentrations of anti-PA Nbs ranged from 0.001 to 10 μg/mL. The error bars represent the standard deviation derived from three independent experiments. **(B)** Thermostability analysis of two libraries-derived anti-PA Nbs. Three Nbs stayed at 37°C for 0, 2, 12, 24, 48, 60 and 72 h were used to perform ELISA. The activity of Nbs never stayed under 37°C was regarded as 100%. Three independent experiments were performed.

To further explore whether the S-anti-PA Nb as good as the I-anti-PA Nb3 and I-anti-PA Nb48 in some features, we compared the Nbs’ reactivity to PA and thermostability. In pMECS, the products of Nb contain both a hexahistidine (His_6_) and hemagglutinin (HA) tag at the C-terminus. His_6_ was used as the affinity tag for the Nbs purification and HA tag provided convenience for the Nbs detection in ELISA. For reactivity comparison, when the 96-well Maxisorp plate was coated with PA, at a concentration of 0.01 μg/mL, the S-anti-PA Nb still showed ability to bind. However, we observed that I-anti-PA Nb3 and I-anti-PA Nb48 could bind with PA even at a lower concentration of 0.005 μg/mL, the binding activity of S-anti-PA Nb was weaker (Figure 5A). In addition, we checked the stability of anti-PA Nbs under heat treatment at 37°C for different incubation time intervals. As shown in Figure 5B, after treatment for 24 h, the S-anti-PA Nb and the two I-anti-PA Nbs could still keep nearly 80% of their binding activity. Similarly, the S-anti-PA Nb displayed lower thermostability with only 20% activity in comparison with 58% activity of the I-anti-PA Nb3 and 68% activity of the I-anti-PA Nb48, after treatment for 48 h. Additionally, after an incubation for 72 h, the S-anti-PA Nb showed almost no activity, however, the two I-anti-PA Nbs still demonstrated about 19% activity (Figure 5B).

### Assessment of Nbs binding to distinct epitopes on PA

We are motivated to test whether the synthetic library-derived Nbs would be promising for further diagnostic applications. As we have isolated two Nbs respectively against PA and NGAL in this study as well as we have obtained three anti-PA Nbs from an immune library in our previous study (Ma et al. [28]), it is interesting to determine whether one of the three I-anti-PA Nbs that could recognize a unique epitope of PA different than the one recognized by the S-anti-PA Nb, which could provide a potential to develop a sandwich ELISA for the detection of PA. By performing the paired experiment, only I-anti-PA Nb48 was identified.

### Nb biotinylation

**Figure 6:**
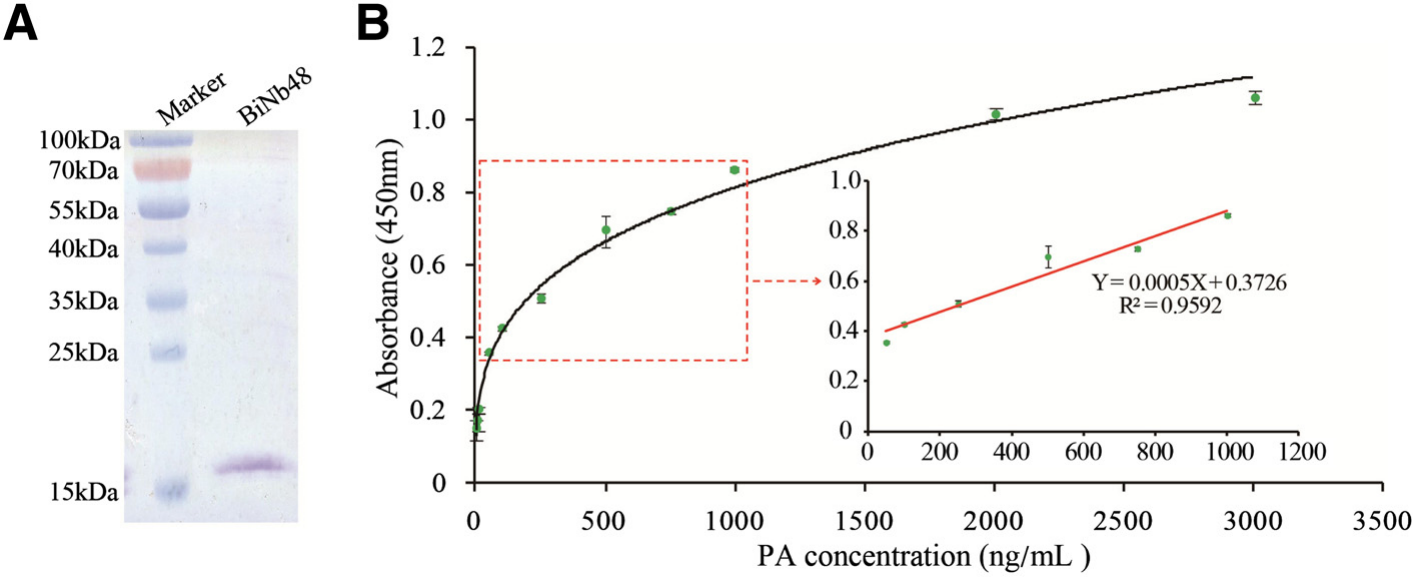
**Detection of PA by a sandwich ELISA based on BiNb48 and the HRP-coupled S-anti-PA Nb. (A)** BiNb48 was eluted into 6 mM biotin solution and analyzed by SDS-PAGE. **(B)** Calibration curve for PA detection. The linear relationship was in the range from 50 to 1000 ng/mL.

According to the results of epitope mapping (data not shown), I-anti-PA Nb48 was chosen to be conjugated with biotin *in vivo* as the capture protein used in the PA detection by ELISA. In order to perform the Nb biotinylation, the VHH genes were sub-cloned into plasmid pBAD17 that contains a biotin acceptor domain and the recombinant plasmids were co-transformed into WK6 cells with another plasmid pBirA to express biotin-conjugated I-anti-PA Nb48. BiNb48 was analyzed by SDS-PAGE (Figure 6A) and it was produced at a final yield of 1 mg/mL culture.

### PA detection by ELISA based on biotin-SA interaction

As shown in Figure 1, BiNb48 were directionally captured by SA coated on BeaverNano™ SA Matrix Coated 96-Well Plate. After PA was recognized and captured by BiNb48, S-anti-PA Nb-HRP was used as the detector. The absorbance at 450 nm displayed a good linearity with the concentrations of PA in the range from 50 to 1000 ng/mL. The linear equation was calculated as Y = 0.0005 X + 0.3726 with an acceptable R^2^ of 0.9592 (Figure 6B), and the LOD was 27.1 ng/mL, illustrating a strong application value of this method for PA detection.

## Discussion

Immunization of camels with various antigens is as straightforward as other animals. However, if the antigens are toxic, of low immunogenicity, limited availability, or nonimmunogenic compounds, immunization will not be feasible for all animals. Such circumstances impose great challenges for the conventional antibody production. Phage display recombinant antibodies could solve these problems by constructing naïve or synthetic libraries with high diversity. Owing to the technical limitations, the maximum size of naïve or synthetic libraries is 8 × 10^9^ CFU/mL [32-35]. Based on a split-mix-split method, a synthetic human scFv antibody library with randomized CDR3 was successfully constructed and applied for isolation of a scFv specific to antigen BHL (anti-CD3 × anti-ovarian carcinoma bispecific antibody) [36]. Naïve phage display Nb libraries have also been reported in several studies [15,37]. Although there is no affinity maturation step like *in vivo* immunization, it is still possible to obtain high affinity Nbs with picomolar affinity [15,37]. However, affinity is also dependent on antigen properties. Subsequent and time-consuming *in vitro* maturation steps can improve affinity [14,15,38]. Phage-displayed Nbs from immune or naïve libraries are made from a natural source of blood. The large sequence diversity in these libraries leads to a large variability in stability, expression level and physical properties. However, *in vitro* approach allows the construction of libraries built on a selected limited set of sequences leading to more consistent and optimized characteristics. Although their lower structural diversity, it is possible to obtain Nbs with high specificity and affinity against any antigen from such libraries. In this study, we chose cAbBCII10 as a universal VHH framework. cAbBCII10 has proved to be a great framework based on its high stability and good expression than the original VHH. Additionally, it is serviceable in the absence of the conserved disulfide bond of the immunoglobulin fold or under reducing conditions, which is a prerequisite for intrabody applications [29]. In order to get great diversity and high quality synthetic Nb library, we introduced random sequence in the CDR3 using degenerate synthetic oligonucleotides. The diversity of natural Nb is mainly due to the sequence variability in the CDR3 and restraining the diversity to this region is sufficient to generate greatly efficient and diverse libraries.

Herein, the random sequences in CDR3 were randomized using trinucleotide cassettes of bacterial preference, which could completely avoid stop and non-sense codons. In addition, the trimer codons for Cys were excluded. After 50 times of high efficiency of transformation, the current size of our library is 1.65 × 10^9^ CFU/mL, which classified as a considerable large library. The overall library size is an important factor for obtaining high affinity clones. As validated by sequencing, almost 100% of randomly picked colonies contained the correct sequences and none Cys or stop codon was found as expected, which meant the functional capacity of the library was also very high. Importantly, non-sense frame-shift mutations were completely absent. However, as it is a synthetic library, as opposed to a naïve library or immune library, it may show to be less functional as they have not been through a natural *in vivo* selection for functionality or affinity maturation [39]. As a consequence, the Nbs isolated from the synthetic library were not exactly as good as that isolated from the immune library in our study. Yet we cannot exclude the possibility that the weaker binding activity and stability of Nbs from the synthesized library is target by target situation. It will be highly possible to isolate Nbs with high affinity and good stability by screening more antigens. Nevertheless, the production of Nbs in *E. coli* is at low cost, it is acceptable to apply the synthetic library-derived Nbs for further diagnostic applications.

We have successfully isolated three Nbs against PA from an immune library with high specificity and affinity in our previous study [28]. Among them, Nb48 recognized a different epitope of PA from that S-anti-PA recognized. Based on the biotin-SA interaction, the S-anti-PA Nb coupled with HRP was used as the detector and the I-anti-PA Nb48 conjugated with biotin was used as the capture protein to detect PA. A linear relation between the absorbance at 450 nm and the concentration of PA was observed in a range from 50 to 1000 ng/mL with a R^2^ of 0.9592, and the LOD is 27.1 ng/mL. The relatively broad consensus reference range of PA for an adult is 120–500 mg/L [22]. Several studies [40,41] have recommended that a concentration of <110 mg/L is high risk, a chief nutritional therapy is necessary; 110–170 mg/L is considered moderate risk, requiring less serious nutritional therapy; and >170 mg/L is of little or no risk. Therefore, reference range values of PA represent a high range for any assay. According to the reference range for PA and the working range we obtained in this study, the samples need to be diluted about 1000-fold before being determined. Therefore, it just needs small amount of serum sample from the patients. The reported methods for determining PA are mainly electrophoresis [42], radioimmunoassay [43], immunonephelometric assays [44], radial immunodiffusion [45], nanogold-labeled immunoresoance scattering spectral assay [46] and ELISA [47]. Electrophoresis analysis has better sensitivity, but it costs long time and it could not assay massive samples at the same time [42]. The relativity of radioimmunoassay is good, but owing to the half-life of isotope, the results are unstable [43]. Immunonephelometric assays were used to measure PA with a working range 1–3 mg/L [44]. Its detection range was not in an order of magnitude with that in this study, as we used an ELISA that its working concentration could not be such high. Radial immunodiffusion is also time-consuming. A nanogold-labeled immunoresoance scattering spectral assay was used to determine 16.67-666.67 ng/mL PA with LOD of 4 ng/mL [46], which was lower than 27.1 ng/mL. However, the process of this assay is a little tedious and it needs more than one special apparatus. Comparatively, ELISA is a simple, straightforward and relatively sensitive approach. An ELISA method based on peroxidase-labeled antibody was used, with a linear range of 1–4 ng per well [47]. According to the reaction volume in the ELISA system, the working range is about 10–40 ng/mL, which was much narrow than 50–1000 ng/mL. Although the LOD in our study is not very low, in consideration to the reference range of PA under different nutritional conditions, it is enough for real PA detection after the patients’ serum samples are diluted 1000-fold, and it needs a very small amount of serum sample, which is more acceptable for the patients.

All together, the trinucleotide cassette-randomized library will be a good source for isolation of some antigen-specific Nbs, which could provide critical support for an immune library or a naïve library. In fact, numerous phage display libraries have been created with many diverse specialized characteristics [48-51] over the past years, the creation of new libraries continues as knowledge development and technology expands.

## Conclusion

In this study, we constructed a large synthetic phage display Nb library based on the conserved VHH framework of cAbBCII10 and diversity was introduced in the CDR3 by randomizing synthetic trinucleotide cassettes. Then Nb against PA or NGAL was successfully selected from this library. Further a sandwich ELISA was fabricated to detect PA with a detection range from 50 to 1000 ng/mL and a detection limit of 27.1 ng/mL based on the HRP-coupled anti-PA Nb obtained from this study and another biotinylated anti-PA Nb isolated from an immune library in our previous study. Thus, this proposed novel synthetic library could provide crucial support to immune library or naïve library in acquirement of specific Nbs, potentially functioning as a great resource for medical diagnostic applications. In addition, we have successfully developed a novel sandwich ELISA to detect PA with a relatively low LOD and wide working range, which is an alternative for the clinically tracing of PA.

## Abbreviations

Nb: Nanobody
Nbs: Nanobodies
VHH: The conserved camel single-domain antibody fragment
CDR3: Complementarity determining region 3
PA: Prealbumin
NGAL: Neutrophil gelatinase-associated lipocalin
IgG: Immunoglobulin G
Fab: Antigen-binding fragment
scFv: Single-chain antibody fragment
HCAbs: Heavy-chain antibodies
CFU: Colony-forming units
HRP: Horseradish peroxidase
Cys: Cysteine
BSA: Bovine serum albumin
FR1: Framework region 1
FR3: Framework region 3
*E. coli*: *Escherichia coli*
ELISA: Enzyme-linked immunosorbent assay
IPTG: Isopropyl-β-d-thiogalactoside
PE-ELISA: Periplasmic extraction ELISA
OD: Optical density
SDS-PAGE: Sodium dodecyl sulfate polyacrylamide gel electrophoresis
AP: Alkaline phosphatase
BNPP: Bis (p-nitrophenyl) phosphate
His_6_: Hexahistidine
HA: Hemagglutinin
CB: Carbonate and bicarbonate
TMB: 3, 3′, 5, 5′-Tetramethylbenzidine
SA: Streptavidin
TB: Terrific broth
kDa: Kilodalton
LOD: Limit of detection
